# Differentiation of high-grade from low-grade diffuse gliomas using diffusion-weighted imaging: a comparative study of mono-, bi-, and stretched-exponential diffusion models

**DOI:** 10.1007/s00234-020-02456-2

**Published:** 2020-05-18

**Authors:** Masaoki Kusunoki, Kazufumi Kikuchi, Osamu Togao, Koji Yamashita, Daichi Momosaka, Yoshitomo Kikuchi, Daisuke Kuga, Nobuhiro Hata, Masahiro Mizoguchi, Koji Iihara, Satoshi O. Suzuki, Toru Iwaki, Yuta Akamine, Akio Hiwatashi

**Affiliations:** 1 Department of Clinical Radiology, Graduate School of Medical Sciences, Kyushu University, 3-1-1 Maidashi, Higashi-ku, Fukuoka, 812-8582 Japan; 2 Department of Neurosurgery, Graduate School of Medical Sciences, Kyushu University, 3-1-1 Maidashi, Higashi-ku, Fukuoka, 812-8582 Japan; 3 Department of Neuropathology, Graduate School of Medical Sciences, Kyushu University, 3-1-1 Maidashi, Higashi-ku, Fukuoka, 812-8582 Japan; 4 Philips Japan, 13-37, Kohnan 2-chome, Minato-ku, Tokyo 108-8507 Japan; 5 Department of Molecular Imaging & Diagnosis, Graduate School of Medical Sciences, Kyushu University, 3-1-1 Maidashi, Higashi-ku, Fukuoka, 812-8582 Japan

**Keywords:** High-grade glioma, Low-grade glioma, Diffusion-weighted imaging, Bi-exponential model, Stretched-exponential model

## Abstract

**Purpose:**

Diffusion-weighted imaging (DWI) plays an important role in the preoperative assessment of gliomas; however, the diagnostic performance of histogram-derived parameters from mono-, bi-, and stretched-exponential DWI models in the grading of gliomas has not been fully investigated. Therefore, we compared these models’ ability to differentiate between high-grade and low-grade gliomas.

**Methods:**

This retrospective study included 22 patients with diffuse gliomas (age, 23–74 years; 12 males; 11 high-grade and 11 low-grade gliomas) who underwent preoperative 3 T-magnetic resonance imaging from October 2014 to August 2019. The apparent diffusion coefficient was calculated from the mono-exponential model. Using 13 *b*-values, the true-diffusion coefficient, pseudo-diffusion coefficient, and perfusion fraction were obtained from the bi-exponential model, and the distributed-diffusion coefficient and heterogeneity index were obtained from the stretched-exponential model. Region-of-interests were drawn on each imaging parameter map for subsequent histogram analyses.

**Results:**

The skewness of the apparent diffusion, true-diffusion, and distributed-diffusion coefficients was significantly higher in high-grade than in low-grade gliomas (0.67 ± 0.67 vs. − 0.18 ± 0.63, 0.68 ± 0.74 vs. − 0.08 ± 0.66, 0.63 ± 0.72 vs. − 0.15 ± 0.73; *P* = 0.0066, 0.0192, and 0.0128, respectively). The 10th percentile of the heterogeneity index was significantly lower (0.77 ± 0.08 vs. 0.88 ± 0.04; *P* = 0.0004), and the 90th percentile of the perfusion fraction was significantly higher (12.64 ± 3.44 vs. 7.14 ± 1.70%: *P* < 0.0001), in high-grade than in low-grade gliomas. The combination of the 10th percentile of the true-diffusion coefficient and 90th percentile of the perfusion fraction showed the best area under the receiver operating characteristic curve (0.96).

**Conclusion:**

The bi-exponential model exhibited the best diagnostic performance for differentiating high-grade from low-grade gliomas.

**Electronic supplementary material:**

The online version of this article (10.1007/s00234-020-02456-2) contains supplementary material, which is available to authorized users.

## Introduction

Gliomas are the most common primary intracranial neoplasms and have various histological grades that reflect malignancy or aggressiveness, according to the World Health Organization (WHO) classification [1]. Distinguishing high-grade gliomas (HGGs; grades III and IV) from low-grade gliomas (LGGs; grades I and II) is important, as their prognoses and therapeutic strategies differ. HGGs are usually treated surgically, followed by concurrent radiation and chemotherapy [2]. HGGs misdiagnosed as LGGs are treated less aggressively than necessary, and vice versa. Therefore, differentiating glioma types before initiating treatment is desirable.

Diffusion-weighted imaging (DWI) provides useful imaging biomarkers for grading gliomas, enabling the quantitative assessment of tumor characteristics without tracer injections. The apparent diffusion coefficient (ADC), which is a useful biomarker reflecting cellular density, is conventionally calculated using two *b*-values (0 and 1000 s/mm^2^) [3]. It has good diagnostic performance in differentiating HGGs from LGGs [3], but there is substantial overlap [4, 5]. Using the ADC in the differential diagnosis may be an oversimplification; it assumes that the only underlying mechanism of observed signal decay is the diffusive motion of water molecules.

Le Bihan et al. [6] proposed using the intravoxel incoherent motion (IVIM) to simultaneously measure perfusion and diffusion. At low *b*-values, the intravoxel motion of water molecules in in vivo tissues is greatly influenced by the microcirculation of blood capillaries (perfusion), whereas at high *b*-values, true diffusive motion predominates, resulting in bi-exponential signal decay. The bi-exponential model for IVIM imaging is useful for differentiating HGGs and LGGs in adults and children [7–9]. Additionally, Bennett et al. introduced the stretched-exponential model [10] to incorporate multicomponent intravoxel diffusion, which leads to non-exponential signal decay. This model enables the measurement of the water diffusion heterogeneity index α, which ranges from 0 to 1 (a value of 1 indicates water diffusion). Accumulating evidence suggests that stretched-exponential DWI is useful for assessing gliomas [11–16] and other tumors, including breast [17], pancreatic [18], hepatic [19], prostate [20], bladder [21], and renal cancers [22].

Histogram analysis is a quantitative technique that has been used for grading gliomas [23, 24]. Kang et al. suggested that histogram-derived ADC values of the whole tumor volume were useful for grading gliomas, while a single ADC value of the regional region-of-interest (ROI) might not effectively reflect the heterogeneous nature of gliomas [24]. To date, two studies [13, 14] have compared the diagnostic performance of mono-, bi-, and stretched-exponential DWI for grading gliomas, using mean ROI values. To the best of our knowledge, a histogram analysis has not been applied to this particular problem. Therefore, the purpose of this study was to compare the imaging parameters obtained from mono-, bi-, and stretched-exponential DWI models for differentiating HGGs from LGGs.

## Methods

This retrospective study was approved by our Institutional Review Board, and the requirement for informed consent was waived.

### Patients

The initial population of 66 patients (55 HGGs and 11 LGGs) met the following inclusion criteria: (1) consecutive patients with histopathologically proven gliomas between October 2014 and August 2019; (2) MRI scans that had been performed within 2 weeks before surgery; (3) patients who had not undergone surgical treatment at the time of the first MRI; and (4) patients for whom the 13 *b*-values of diffusion imaging were acquired. Of the 66 patients, 44 were excluded to create a cohort of equal numbers of consecutive HGG and LGG patients (for statistical simplicity as well as preventing bias from overrepresentation of HGGs; all data [*N* = 55 HGG patients] is shown in Online Resources 4 and 5). In total, 22 patients, including 11 consecutive patients with HGGs (range, median age; 31–71, 65 years; 4 males and 7 females) treated from June 2018 to October 2018, and 11 with LGGs (23–74, median age; 45 years; 8 males and 3 females), were enrolled from October 2014 to August 2019. The inclusion and exclusion criteria are summarized in Online Resource 1. The period of enrollment differed between patients with HGGs and LGGs, since we included the most recent 11 consecutive patients with HGGs.

### Histopathologic diagnosis

The patients’ demographics and pathological diagnoses, based on the 2016 WHO classification [1], are as follows: the 11 HGGs included 7 glioblastomas (isocitrate dehydrogenase (IDH)-wild type; WHO grade IV; age, 48–71 years; 3 males and 4 females); 1 diffuse midline glioma (H3 K27M-mutant type; WHO grade III; age, 31 years; female); 2 anaplastic astrocytomas (IDH-mutant type; WHO grade III; age, 32 and 71 years; 1 male and 1 female); and 1 anaplastic oligodendroglioma (IDH-mutant type; WHO grade III; age, 47 years; female). The 11 LGGs included 5 diffuse astrocytomas (IDH-mutant type; WHO grade II; age, 23–45 years; 4 males and 1 female); 4 diffuse astrocytomas (IDH-wild type; WHO grade II; age, 29–74 years; 3 males and 1 female); and 2 oligodendrogliomas (IDH-mutant type, WHO grade II; age, 49 and 73 years; 1 male and 1 female).

### MRI

MRI was performed as previously described [9] using 3 T MR scanners (Achieva 3.0 T TX or Ingenia 3.0 T CX; Philips Healthcare, Best, The Netherlands) with 8- or 15-channel head coils. DWI was obtained in the axial plane using a two-dimensional single-shot spin-echo echo-planar imaging diffusion sequence. We used 13 *b*-values (0, 10, 20, 30, 50, 80, 100, 200, 300, 400, 600, 800, and 1000 s/mm^2^) in three orthogonal directions. At our institution, preoperative imaging of patients with brain tumors routinely uses the same 13 *b*-values. The other MRI parameters were as follows: repetition time/echo time, 2500/70 ms; matrix, 128 × 126 (reconstructed to 256 × 256); number of excitations, 1; section thickness/gap, 5/1 mm; FOV, 230 × 230 mm^2^; number of sections, 11; sensitivity encoding factor, 1.5; and total scan time, 2 m 7 s. Standard MR sequences (T1-, and T2-weighted images, fluid attenuated inversion recovery, and contrast-enhanced T1-weighted images) were obtained for diagnostic purposes. DWI was acquired before contrast agent injection. Imaging data were used for mono-, bi-, and stretched-exponential DWI models for subsequent image analysis. No denoising or co-registration for the DWI data were performed before analysis.

### Image analysis

From the acquired images, model fitting was performed using image analyzer software (diffusion analysis software, EXPRESS 2.0; Philips Healthcare) [25]. The mono-exponential DWI provides the ADC values using the formula:

1 $$ \frac{{\mathrm{SI}}_{1000}}{{\mathrm{SI}}_0}=\exp\ \left(-b\times \mathrm{ADC}\right) $$

where SI_0_ corresponds to the signal intensity without diffusion weighting (*b* = 0 s/mm^2^) and SI_1000_ is the signal intensity at *b* = 1000 s/mm^2^. For bi-exponential DWI data analysis, the bi-exponential model was defined by the following equation:

2 $$ \frac{{\mathrm{SI}}_{\mathrm{b}}}{{\mathrm{SI}}_0}=f\times \exp\ \left(-b\times {D}^{\ast}\right)+\left(1-f\right)\times \exp\ \left(-b\times D\right) $$

where SI_b_ is the signal intensity acquired with different *b*-values, *f* is the perfusion fraction, D* is the pseudo-diffusion coefficient, and *D* is the true-diffusion coefficient. The bi-exponential DWI model is based on the fit of three parameters. First, the *D* was determined from data with higher *b*-values (b = 300, 400, 600, 800, and 1000 s/mm^2^). When high *b*-values were used and the IVIM component was negligible, the following least-squares curve fit was used:

3 $$ \frac{{\mathrm{SI}}_{\mathrm{b}}}{{\mathrm{SI}}_0}=\exp\ \left(-b\times D\right) $$

Second, the segmented method was used to calculate *f* according to the following equation:

4 $$ f=\left({\mathrm{SI}}_0-{\mathrm{SI}}_{\mathrm{inter}}\right)/{\mathrm{SI}}_0 $$

Here, SI_inter_ is the intersection point of the *y*-axis and line through ln SI_300_ and ln SI_1000_. Third, D* was derived from the mono-exponential fit to Eq. (2).

The stretched-exponential DWI model is defined as follows:

5 $$ \frac{{\mathrm{SI}}_{\mathrm{b}}}{{\mathrm{SI}}_0}=\exp\ \left\{-{\left(b\times \mathrm{DDC}\right)}^{\alpha}\right\} $$

where DDC is the distributed-diffusion coefficient, and *α* is the heterogeneity index. All 13 *b*-values were used to provide the best-fit parameter values for DDC and *α* simultaneously.

Two radiologists carefully evaluated each image on consensus, identified the T2-prolonged region where tumors existed, and drew ROIs on each tumor map for subsequent analysis. We used the single maximum section of each tumor for the ROI analysis. When placing the ROIs for each map, we carefully avoided cortical gray matter, which affects the *f* value. ROIs were copied on each parameter map to obtain pixel-by-pixel values for histogram analyses. The 10th, 25th, 50th (median), 75th, and 90th percentiles, as well as the mean, skewness, and kurtosis, of each parameter were recorded from the histograms. Note that our definition of an ROI may include edematous or necrotic regions within the tumor.

### Statistical analysis

The percentiles, as well as the mean, skewness, and kurtosis of each parameter (i.e., ADC, *D*, D*, *f*, DDC, and *α*), were compared between HGGs and LGGs using the Mann-Whitney *U* test. The diagnostic performance of each model parameter was evaluated by receiver operating characteristic (ROC) curve analysis. Combinations of two parameters from bi- and stretched-exponential DWI models were also assessed. Two independent areas under the curves (AUCs) were compared using the method developed by Delong et al. [26]. All statistical analyses were performed using commercial software programs (JMP, version 14.0.0; SAS Institute, Cary, NC, USA; Prism 7.0, GraphPad Software, La Jolla, CA, USA). *P* values < .05 were considered statistically significant.

## Results

### Histogram analysis

**Fig. 1:**
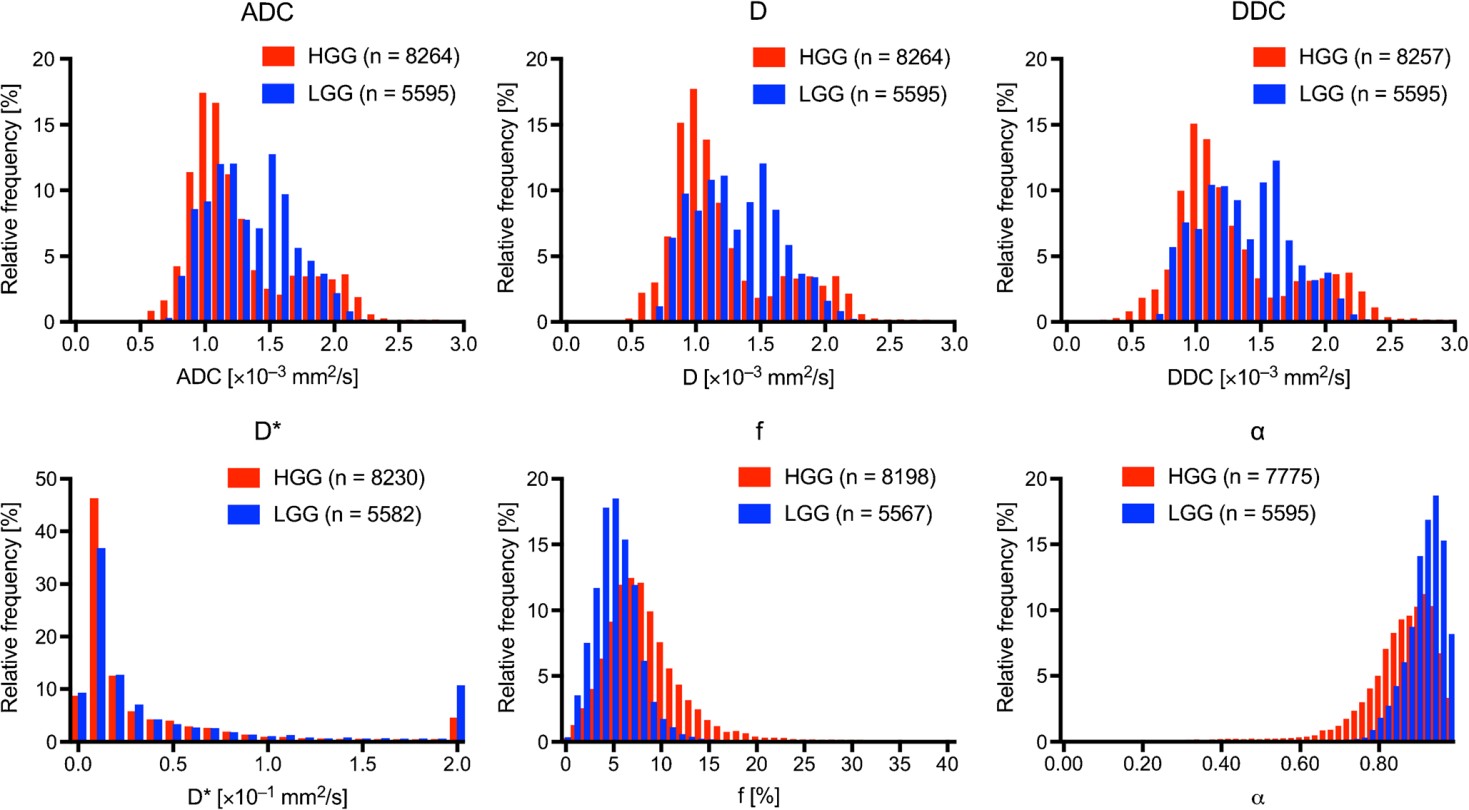
Normalized histograms of each parameter Diffusion-related parameters (ADC, D, and DDC) and the heterogeneity index *α* from HGGs exhibit a slight leftward shift (“+” skewness) relative to those from LGGs, while the *f* from HGGs shows a slight rightward shift (“−” skewness) relative to that from LGGs. There is no difference in the D* between the two types of gliomas. *α* heterogeneity index, ADC apparent diffusion coefficient, *D* true-diffusion coefficient, DDC distributed diffusion coefficient, D* pseudo-diffusion coefficient, *f* perfusion fraction, HGG high-grade diffuse glioma, LGG low-grade diffuse glioma

Figure 1 shows the normalized histograms of each parameter over all pixels in tumor ROIs. The ADC, *D*, DDC, and *α* from HGGs all exhibited a slight leftward shift (“+” skewness) relative to those from LGGs, while the *f* from HGGs showed a slight rightward shift (“−” skewness) relative to that from LGGs. No significant difference in D* between the two types of gliomas was observed.

### Differentiation of HGGs and LGGs using parameter measurements

Table 1 shows the parameter measurements in the HGGs and LGGs. The 10th, 25th, 50th, 75th, and 90th percentiles of each parameter, as well as skewness and kurtosis, were all recorded from the histograms; the most useful values of each parameter are shown in Table 1. The entire set of our results can be found in Online Resource 2. For diffusion-related parameters, the skewness of ADC (ADC_skw_), *D*_skw_, and DDC_skw_ were significantly higher in HGGs than in LGGs (0.67 ± 0.67 vs. − 0.18 ± 0.63, 0.68 ± 0.74 vs. − 0.08 ± 0.66, 0.63 ± 0.72 vs. − 0.15 ± 0.73; *P* = 0.007, 0.002, and 0.01, respectively). The 10th percentile of *α* (α10) was significantly lower in HGGs than in LGGs (0.77 ± 0.08 vs. 0.88 ± 0.04; *P* < 0.001). The 90th percentile of the perfusion fraction (f90) was significantly higher in HGGs than that in LGGs (12.64 ± 3.44 vs. 7.14 ± 1.70%; *P* < 0.001). There was no significant difference in D* for differentiating between the two groups of gliomas (*P* = 0.30 in the kurtosis of D*).

**Table 1:** Comparison of parameters between high- and low-grade diffuse gliomas

Parameters	High-grade glioma	Low-grade glioma	*P* value
ADC_skw_	0.67 (0.22–1.12)	− 0.18 (− 0.60–0.24)	0.007^a^
D_skw_	0.68 (0.18–1.18)	− 0.08 (− 0.52–0.37)	0.02^a^
DDC_skw_	0.63 (0.15–1.12)	− 0.15 (− 0.64–0.33)	0.01^a^
α10	0.77 (0.72–0.83)	0.88 (0.86–0.91)	< 0.001^a^
D*_kur_	12.85 (− 1.69–27.40)	13.13 (− 5.09–31.35)	0.30^a^
f90 (%)	12.64 (10.33–14.95)	7.14 (6.00–8.28)	< 0.001^a^

Data are expressed as mean values and 95% confidence intervals

*α* heterogeneity index, *ADC* apparent diffusion coefficient, *D* true-diffusion coefficient, D* pseudo-diffusion, *DDC* distributed-diffusion coefficient, *f* perfusion fraction, *kur* kurtosis, *skw* skewness

^a^Mann-Whitney *U* test

### ROC analysis

Table 2 shows the diagnostic performance and most useful values of the investigated parameters in differentiating HGGs and LGGs. The entire set of our results can be found in Online Resource 3. In the single-parameter analysis, the f90 showed one of the highest diagnostic performances (AUC = 0.96), with an optimal diagnosis cutoff value of 9.1%. The α10 also showed good diagnostic performance (AUC = 0.91, cutoff value 0.85).

**Table 2:** Diagnostic performance of parameters in differentiating between high- and low-grade diffuse gliomas

Parameters	Sensitivity (%)	Specificity (%)	Accuracy (%)	Positive predictive value (%)	Negative predictive value (%)	Cutoff value	AUC
ADC_skw_	72.7	90.9	81.8	88.9	76.9	> 0.38	0.83
*D*_skw_	63.6	90.9	77.3	87.5	71.4	> 0.66	0.79
D*_skw_	81.8	54.6	68.2	64.3	75.0	> 1.50	0.62
f90 (%)	100	90.9	95.5	91.7	100	> 9.1	0.96
DDC_skw_	63.6	90.9	77.3	87.5	71.4	> 0.48	0.81
α10	81.8	90.9	86.4	90.0	83.3	≤ 0.85	0.91
D10 + f90	100	90.9	95.5	91.7	100	≤ 0.87, > 9.1	0.96
DDC_skw_ + α10	81.8	90.9	86.4	90.0	83.3	≤ − 0.64, ≤ 0.76	0.93

*α* heterogeneity index, *ADC* apparent diffusion coefficient, *AUC* area under the curve, *D* true-diffusion coefficient, D* pseudo-diffusion coefficient, *DDC* distributed-diffusion coefficient, *f* perfusion fraction

In the combined-parameters analysis, the combination of the D10 and f90 from the bi-exponential DWI model also showed one of the highest diagnostic performances (AUC = 0.96, cutoff values 0.87 × 10^−3^ mm^2^/s and 9.1% for D10 and f90, respectively). The combination of the DDC_skw_ and α10 from the stretched-exponential DWI model exhibited good diagnostic performance (AUC = 0.93, cutoff values − 0.64 and 0.76 for DDC_skw_ and α10, respectively); however, no significant differences were detected between these combinations (D10 + f90, DDC_skw_ + α10) and f90 (*P* = 1.000, 0.2059, respectively) or α10 (*P* = 0.2894, 0.6603, respectively).

**Fig. 2:**
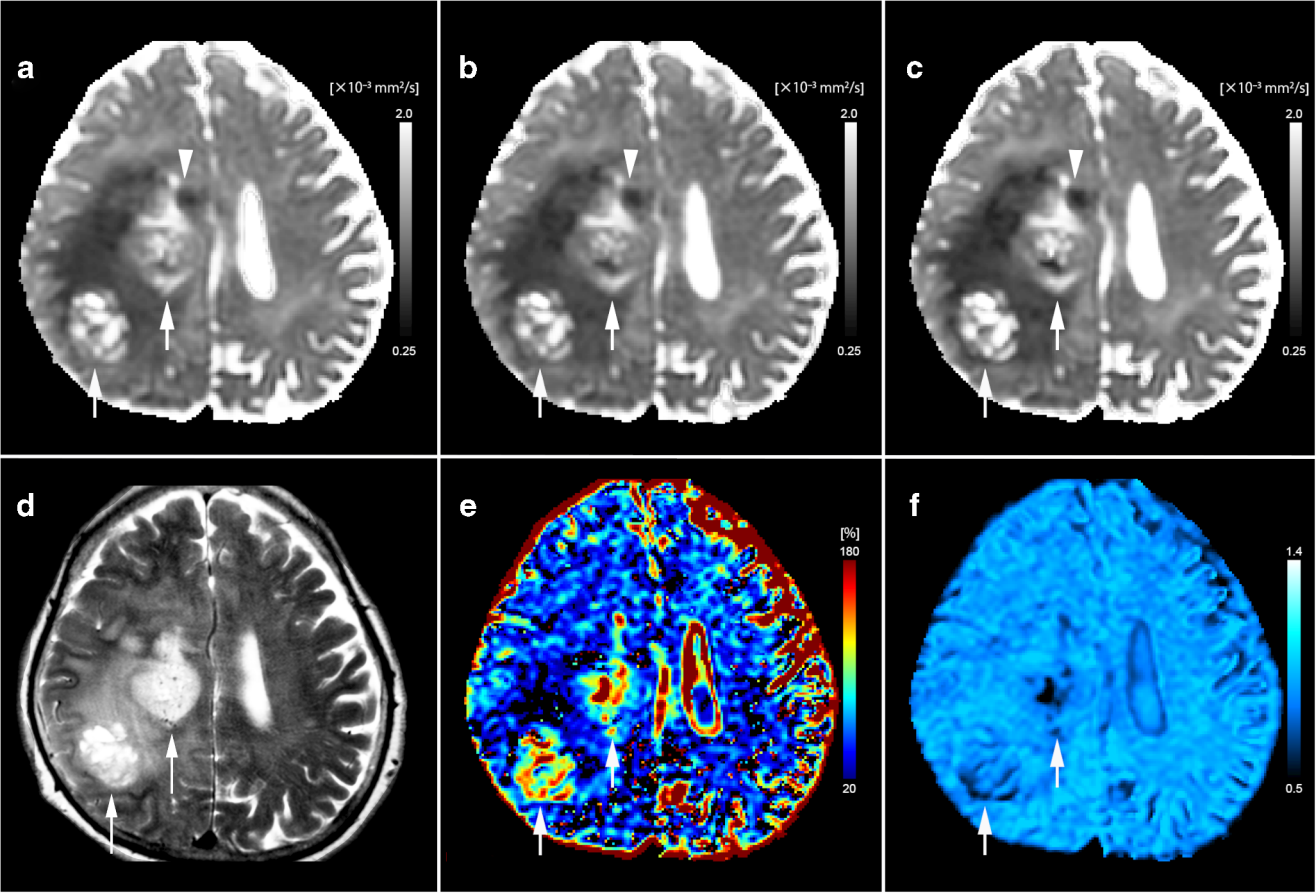
Images from a 70-year-old woman with histologically proven glioblastoma, IDH-wild type (WHO grade IV). Diffusion-related coefficient maps show heterogeneous masses of low values (**a** ADC map; **b***D* map; **c** DDC map; 0.79, 0.63, 0.60 × 10^−3^ mm^2^/s, respectively) in the right parietal lobe and centrum semiovale (arrows). There is a substantial restricted diffusion in the right frontal deep white matter (arrowheads). **d** The T2-weighted image shows heterogeneously hyperintense foci in the mass. **e** The *f* map shows a high value (15.6%) in the lesion. **f** The *α* map shows a low value (0.65) in the lesion. *α* heterogeneity index, ADC apparent diffusion coefficient, *D* true-diffusion coefficient, DDC distributed diffusion coefficient, *f* perfusion fraction, IDH isocitrate dehydrogenase, WHO World Health Organization

**Fig. 3:**
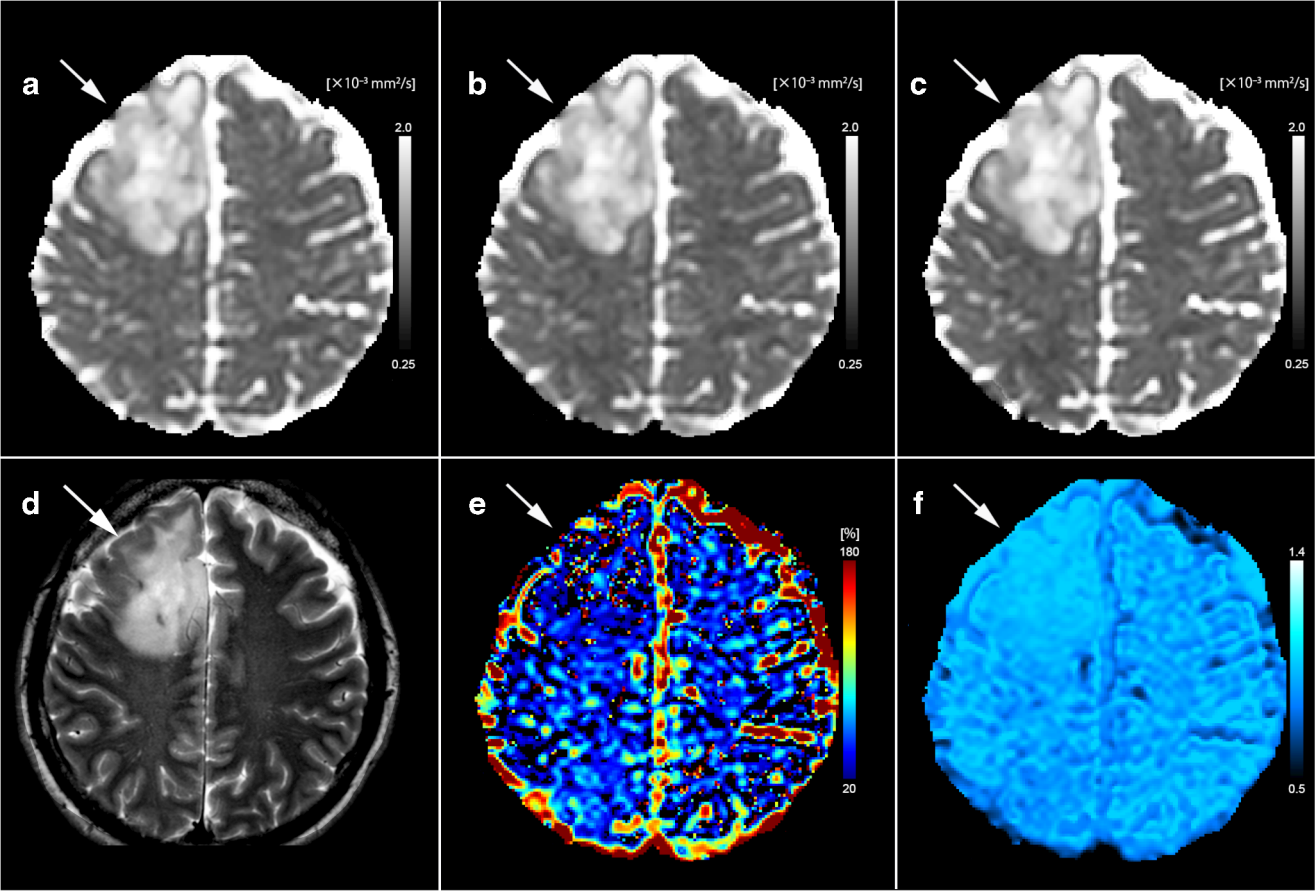
Images from a 49-year-old woman with histologically proven oligodendroglioma, IDH-mutant type (WHO grade II). Diffusion-related coefficient maps show a heterogeneous mass of high values (**a** ADC map, **b***D* map, **c** DDC map; 1.04, 1.00, 1.05 × 10^−3^ mm^2^/s, respectively) in the right frontal lobe (arrows). **d** The T2-weighted image shows heterogeneously hyperintense mass. **e** The *f* map shows a low value (6.2%) in the lesion. **f***α* map shows a high value (0.89) in the lesion. *α* heterogeneity index, ADC apparent diffusion coefficient, *D* true-diffusion coefficient, DDC distributed diffusion coefficient, *f* perfusion fraction, IDH isocitrate dehydrogenase, WHO World Health Organization

Figures 2 and 3 show representative images from patients with grades IV (glioblastoma) and II (oligodendroglioma) gliomas, respectively.

## Discussion

We examined histogram-based parameters from mono-, bi-, and stretched-exponential DWI models to compare their diagnostic performance in distinguishing HGGs from LGGs. The ADC_skw_, D_skw_, and DDC_skw_ were found to be useful diffusion parameters, providing good sensitivity and moderate specificity. Previous reports revealed that the ADC correlates well with cell density [27, 28]. Dense cells provide greater restriction of water movement, presumably due to increased cell membranes and intracellular space fraction. Accordingly, ADC measurements have been utilized to assess cell density in various tumors. The ADC calculated from two *b*-values (0 and 1000 s/mm^2^) decreases with glioma grade based on their histological features, including cell proliferation [29–31]. In the bi-exponential model, *D* was fitted only using high *b*-values, while the ADC and DDC were not. Therefore, *D* reflects cell density more effectively than ADC and DDC by eliminating the effect of perfusion. The fast blood flow of the microcirculation observed at low *b*-values would yield slightly higher ADC and DDC values relative to the *D* value, as seen in our results. Furthermore, skewness represents a measure of asymmetry of the probability distribution. If a histogram has an elongated tail on the left side of the mean, it is negatively skewed; conversely, if a histogram has an elongated tail on the right side of the mean, it is positively skewed. In the present study, diffusion-related parameters from HGGs exhibited a slight leftward shift, meaning they were positively skewed. Therefore, HGGs had a greater frequency of pixels with lower diffusion-related parameters than the mean, compared with LGGs. While various components, such as edematous and necrotic tissues, exist within the HGGs, the result indicates the presence of more regions of higher density cells (i.e., tumor cells), showing the asymmetric leftward shift of the histograms. King et al. [32] revealed that there is a significant intra-treatment decrease in skewness in patients with head and neck squamous cell carcinomas. Our diffusion parameter results are consistent with those of previous studies [8, 9, 32].

The D* was not helpful in glioma grading, as reported previously [8, 9, 33]. The D* is a parameter of the fast component of the bi-exponential model and is sensitive to glioma microcirculation. Its poor reproducibility could be due to cardiac motion [34]. Cardiac-gating techniques may improve D* assessment in tumors [35].

Our results indicated that a high percentile of *f* (i.e., f90) from the bi-exponential model is a powerful imaging biomarker for differentiating HGGs from LGGs. One aspect of glioma malignancy is angiogenesis, a key feature of the histopathological assessment in the WHO grading system. In the bi-exponential model, the fast component at low *b*-values is assumed to reflect the blood flow of the microcirculation, while the slow component at high *b*-values is governed by the pure diffusive motion of water molecules. The f90 of HGGs was significantly higher than that of LGGs, in agreement with previous studies [7–9, 36]. However, Bai et al. reported that the *f* was significantly lower in HGGs than in LGGs [13], which contradicts our results. Further investigations are needed to resolve this issue.

A low percentile of the heterogeneity index *α* (i.e., α10) from the stretched-exponential model is also useful for differentiating HGGs from LGGs. The *α* measures how much the signal decay deviates from the mono-exponential decay within a voxel. A value of *α* close to one indicates high homogeneity in apparent diffusion. Lower values of *α* indicate high heterogeneity, namely, the multiple-component decay from multiple-apparent diffusion of water molecules. In the WHO glioma grading system, HGGs require the presence of histopathological features such as anaplasia of glial cells, mitotic activity, and microvascular proliferation and/or necrosis, while LGGs require at most cytological atypia alone [1]. Therefore, HGGs would include more distinct structural components within the voxel and are microscopically more heterogeneous than are LGGs, providing lower values of *α*. Few studies have applied the *α* to brain tumors. Kwee et al. demonstrated that the *α* of HGGs is significantly different from that of normal brain structures [11]. Other studies showed that the *α* of HGGs is significantly lower than that of LGGs [13, 14, 16], consistent with our findings.

In the combined-parameters analysis, the combination of the D10 and f90 showed the best diagnostic performance of all the parameters investigated in our study. In combination, these two parameters can be used to evaluate different pathologic features, vascularity, and cell density in gliomas. Some glioblastomas exhibit high vascularity and low cell density due to intratumoral necrosis. Thus, the bi-exponential DWI model is particularly useful in such cases because it can simultaneously evaluate both, as well as perfusion parameters, in the same anatomical space, which provides an advantage over the single-parameter analysis of the mono-exponential model. Although the AUC value for the combined DDCs_kw_ and α10 was lower than that for the combined D10 and f90, it achieved a good diagnostic performance. The stretched-exponential model enables the simultaneous measurement of diffusion and heterogeneity. Other tumors with different histological features would show different patterns of diffusion, vascularity, and heterogeneity. It would be useful to assess which model is most effective for differentiating tumor malignancies.

This study has some limitations. First, the number of patients was small. Moreover, we matched the number of patients with HGGs and LGGs for statistical simplicity in this pilot study based on preliminary data. It would be desirable to proceed with a subsequent study with a larger number of patients. Second, the study design was retrospective in nature; thus, a patient selection bias could not be completely eliminated. Third, we did not evaluate *b*-values > 1000 s/mm^2^ in the measurements, which is unlike previous studies; however, an optimal upper *b*-value limit for brain tumors has not been determined. Fourth, we did not include the whole tumor volume in the histogram analysis. Instead, we used the maximum section of the glioma, with its boundary defined by hyperintensity on T2-weighted images. However, in a previous study, whole-volume histogram analysis did not yield more accurate results than single-slice methods and took longer to complete [37]. In the ROI analysis, edematous or necrotic regions were included. However, identifying these regions would be a subjective process. Instead, we employed a histogram analysis with ROIs of all T2-prolonged areas of each tumor, which we believe is a more objective method, in the current study.

## Conclusions

The mono-, bi-, and stretched-exponential DWI models provided useful imaging biomarkers related to essential histological features for the differentiation of gliomas using histogram analysis. In particular, the bi-exponential model exhibited the best diagnostic performance for differentiating HGGs from LGGs either by evaluation of the perfusion parameter or both the diffusion and perfusion parameters simultaneously, thereby providing a helpful noninvasive diagnostic method for grading gliomas.

## Electronic supplementary material

ESM 1 (PDF 616 kb)
