# Supplementary material for: Differentiation of high-grade from low-grade diffuse gliomas using diffusion-weighted imaging: a comparative study of mono-, bi-, and stretched-exponential diffusion models
Source: Neuroradiology. 2020 May 18;62(7):815–23. doi: 10.1007/s00234-020-02456-2 (PMC7311374; doi:10.1007/s00234-020-02456-2)
Supplement: Supplementary file 1 — (PDF 616 kb) [file 234_2020_2456_MOESM1_ESM.pdf]

Differentiation of High-Grade from Low-Grade Diffuse Gliomas Using Diffusion-Weighted Imaging: A Comparative Study of Mono-, Bi-, and Stretched-Exponential Diffusion Models

Journal name: Neuroradiology

Masaoki Kusunoki, MD, PhD[1], Kazufumi Kikuchi, MD, PhD[1], Osamu Togao, MD, PhD[1], Koji Yamashita, MD, PhD[1], Daichi Momosaka, MD[1], Yoshitomo Kikuchi, MD[1], Daisuke Kuga, MD, PhD[3], Nobuhiro Hata, MD, PhD[3], Masahiro Mizoguchi, MD, PhD[3], Koji Iihara, MD, PhD[3], Satoshi O Suzuki, MD, PhD[4], Toru Iwaki, MD, PhD[4], Yuta Akamine, MSc[5], Akio Hiwatashi, MD, PhD[2]

Departments of [1]Clinical Radiology, [2]Molecular Imaging & Diagnosis, [3]Neurosurgery, and [4]Neuropathology
Graduate School of Medical Sciences, Kyushu University, Fukuoka, Japan
3-1-1 Maidashi, Higashi-ku, Fukuoka 812-8582, Japan
[5]Philips Japan, 13-37, Kohnan 2-chome, Minato-ku, Tokyo, 108-8507, Japan

Correspondence:
Kazufumi Kikuchi, MD, PhD
Department of Clinical Radiology
Graduate School of Medical Sciences
Kyushu University
3-1-1 Maidashi, Higashi-ku, Fukuoka 812-8582, Japan
Tel: +81-92-642-5695
Fax: +81-92-642-5708
E-mail: kkikuchi@radiol.med.kyushu-u.ac.jp

Online Resource 1. Inclusion and exclusion criteria and study flow diagram

Inclusion criteria:

1. Consecutive patients with histopathologically proven glioma treated between October 2014 and October 2018
2. MRI scans that had been obtained within 1 month before surgery
3. Patients who had not undergone surgical treatment at the time of the first MRI
4. Patients who had undergone the 3 types of exponential diffusion imaging

Initial population

- 66 patients
  55 high-grade gliomas (HGGs)
  11 low-grade gliomas (LGGs)

Exclusion criterion:

1. Exclusion (N = 44) to create HGG and LGG cohorts with an equal number of consecutive patients for statistical simplicity

Study population

- 22 patients
  11 HGGs
  11 LGGs

Online Resource 2. Comparison of parameters between high- and low-grade diffuse gliomas

**Parameters**	**High-grade diffuse glioma**	**Low-grade diffuse glioma**	***P*-value**
ADC10 [$\times 10^{-3}$ mm^2/s]	0.98 (0.81–1.15)	1.21 (1.02–1.40)	0.0557[a]
ADC25 [$\times 10^{-3}$ mm^2/s]	1.07 (0.90–1.25)	1.30 (1.09–1.51)	0.0652[a]
ADC50 [$\times 10^{-3}$ mm^2/s]	1.19 (1.00–1.38)	1.39 (1.16–1.62)	0.1513[a]
ADC75 [$\times 10^{-3}$ mm^2/s]	1.33 (1.12–1.55)	1.47 (1.23–1.71)	0.5190[a]
ADC90 [$\times 10^{-3}$ mm^2/s]	1.47 (1.25–1.70)	1.54 (1.29–1.78)	0.6994[a]
ADC_{skw}	0.67 (0.22–1.12)	–0.18 (–0.60–0.24)	0.0066[a]
ADC_{kur}	1.01 (–0.10–2.12)	0.05 (–0.49–0.59)	0.3000[a]
D10 [$\times 10^{-3}$ mm^2/s]	0.92 (0.74–1.10)	1.19 (0.99–1.38)	0.0400[a]
D25 [$\times 10^{-3}$ mm^2/s]	1.02 (0.84–1.20)	1.27 (1.06–1.49)	0.0557[a]

D50 [× 10^{-3} mm^2/s]	1.14 (0.94–1.34)	1.36 (1.12–1.59)	0.1051[a]
D75 [× 10^{-3} mm^2/s]	1.29 (1.07–1.52)	1.44 (1.19–1.68)	0.3653[a]
D90 [× 10^{-3} mm^2/s]	1.44 (1.20–1.67)	1.51 (1.26–1.77)	0.6522[a]
D_{skw}	0.68 (0.18–1.18)	–0.08 (–0.52–0.37)	0.0192[a]
D_{kur}	1.14 (–0.10–2.38)	0.15 (–0.38–0.68)	0.2703[a]
D*10 [× 10^{-1} mm^2/s]	0.06 (0.04–0.07)	0.06 (0.04–0.08)	0.9605[a]
D*25 [× 10^{-1} mm^2/s]	0.08 (0.06–0.11)	0.11 (0.05–0.18)	0.6115[a]
D*50 [× 10^{-1} mm^2/s]	0.15 (0.09–0.21)	0.27 (0.05–0.50)	0.8584[a]
D*75 [× 10^{-1} mm^2/s]	0.35 (0.18–0.52)	0.59 (0.15–1.04)	0.7598[a]
D*90 [× 10^{-1} mm^2/s]	0.88 (0.39–1.39)	1.04 (0.45–1.62)	0.7594[a]
D^*_{skw}	2.71 (1.17–4.26)	2.49 (0.65–4.33)	0.3653[a]

D^*_{kur}	12.85 (–1.69–27.40)	13.13 (–5.09–31.35)	0.3000[a]
f10 [%]	4.86 (3.42–6.31)	2.71 (1.91–3.50)	0.0120[a]
f25 [%]	6.40 (4.74–8.05)	3.77 (2.97–4.56)	0.0051[a]
f50 [%]	8.36 (6.37–10.35)	4.94 (4.07–5.80)	0.0005[a]
f75 [%]	10.47 (8.19–12.76)	6.02 (5.04–7.01)	<0.0001[a]
f90 [%]	12.64 (10.33–14.95)	7.14 (6.00–8.28)	<0.0001[a]
f_{skw}	1.053 (0.47–1.64)	0.25 (–0.10–0.59)	0.0128[a]
f_{kur}	12.64 (10.33–14.95)	0.57 (–0.35–1.49)	0.0192[a]
DDC10 [$\times 10^{-3}$ mm^2/s]	0.97 (0.75–1.20)	1.24 (1.02–1.45)	0.0729[a]
DDC25 [$\times 10^{-3}$ mm^2/s]	1.09 (0.88–1.30)	1.33 (1.09–1.56)	0.0652[a]
DDC50 [$\times 10^{-3}$ mm^2/s]	1.23 (1.00–1.47)	1.42 (1.17–1.67)	0.2357[a]

DDC75 [$\times 10^{-3}$ mm^2/s]	1.42 (1.16–1.68)	1.51 (1.25–1.77)	0.6994[a]
DDC90 [$\times 10^{-3}$ mm^2/s]	1.60 (1.32–1.88)	1.58 (1.32–1.84)	>0.9999[a]
DDC_{skw}	0.63 (0.15–1.12)	–0.15 (–0.64–0.33)	0.0128[a]
DDC_{kur}	1.03 (0.09–1.97)	0.17 (–0.56–0.90)	0.1330[a]
α10	0.77 (0.72–0.83)	0.88 (0.86–0.91)	0.0004[a]
α25	0.82 (0.78–0.86)	0.91 (0.88–0.93)	0.0024[a]
α50	0.86 (0.83–0.90)	0.92 (0.90–0.95)	0.0083[a]
α75	0.90 (0.88–0.93)	0.94 (0.92–0.96)	0.0222[a]
α90	0.93 (0.91–0.95)	0.96 (0.94–0.97)	0.0879[a]
α_{skw}	–0.80 (–1.27– –0.34)	–0.49 (–1.03–0.06)	0.3653[a]
α_{kur}	1.42 (–0.002–2.83)	1.10 (–0.28–2.49)	0.6994[a]

Data are expressed as mean values and 95% confidence intervals.

α, heterogeneity index; *ADC*, apparent diffusion coefficient; *D*, true-diffusion coefficient; *DDC*, distributed-diffusion coefficient; *f*, perfusion fraction; *kur*, kurtosis; *skw*, skewness

[a]Mann-Whitney U-test

Online Resource 3. Diagnostic performance of parameters in differentiating between high- and low-grade diffuse gliomas

**Parameters**	**Sen [%]**	**Spe [%]**	**Acc [%]**	**PPV [%]**	**NPV [%]**	**Cutoff value**	**AUC**
ADC10 [$\times 10^{-3}$ mm^2/s]	81.8	81.8	81.8	81.8	81.8	≤0.94	0.74
ADC25 [$\times 10^{-3}$ mm^2/s]	81.8	72.7	77.3	75.0	80.0	≤1.05	0.74
ADC50 [$\times 10^{-3}$ mm^2/s]	72.7	72.7	72.7	72.7	72.7	≤1.15	0.69
ADC75 [$\times 10^{-3}$ mm^2/s]	81.8	54.6	68.2	64.3	75.0	≤1.30	0.59
ADC90 [$\times 10^{-3}$ mm^2/s]	81.8	54.6	68.2	64.3	75.0	≤1.49	0.55
ADC_{skw}	72.7	90.9	81.8	88.9	76.9	>0.38	0.83
ADC_{kur}	27.3	100	63.6	100	57.9	>2.19	0.64
D10 [$\times 10^{-3}$ mm^2/s]	81.8	81.8	81.8	81.8	81.8	≤0.90	0.76
D25 [$\times 10^{-3}$ mm^2/s]	81.8	81.8	81.8	81.8	81.8	≤0.96	0.74

D50 [× 10^{-3} mm^2/s]	81.8	72.7	77.3	75.0	80.0	≤1.12	0.71
D75 [× 10^{-3} mm^2/s]	81.8	63.6	72.7	69.2	77.8	≤1.24	0.62
D90 [× 10^{-3} mm^2/s]	81.8	54.6	68.2	64.3	75.0	≤1.41	0.56
D_{skw}	63.6	90.9	77.3	87.5	71.4	>0.66	0.79
D_{kur}	27.3	100	63.6	100	57.9	>1.34	0.64
D*10 [× 10^{-1} mm^2/s]	81.8	36.4	59.1	56.3	66.7	>0.05	0.49
D*25 [× 10^{-1} mm^2/s]	81.8	36.4	59.1	56.3	66.7	>0.09	0.57
D*50 [× 10^{-1} mm^2/s]	54.6	63.6	59.1	60.0	58.3	>0.11	0.52
D*75 [× 10^{-1} mm^2/s]	81.8	45.5	63.6	60.0	71.4	>0.57	0.54
D*90 [× 10^{-1} mm^2/s]	72.7	45.5	59.1	57.1	62.5	>1.09	0.54
D^*_{skw}	81.8	54.6	68.2	64.3	75.0	≤1.51	0.62

D^*_{kur}	100	9.1	54.6	52.4	100	>74.01	0.36
f10 [%]	72.7	90.9	81.8	88.9	76.9	>4.30	0.81
f25 [%]	81.8	81.8	81.8	81.8	81.8	>5.00	0.84
f50 [%]	81.8	90.9	86.4	90.0	83.3	>6.40	0.91
f75 [%]	90.9	90.9	90.9	90.9	90.9	>7.30	0.95
f90 [%]	100	90.9	95.5	91.7	100	>9.10	0.96
f_{skw}	81.8	72.7	77.3	75.0	80.0	≤0.49	0.81
f_{kur}	81.8	81.8	81.8	81.8	81.8	>0.80	0.79
DDC10 [$\times 10^{-3}$ mm^2/s]	81.8	81.8	81.8	81.8	81.8	≤0.92	0.73
DDC25 [$\times 10^{-3}$ mm^2/s]	81.8	81.8	81.8	81.8	81.8	≤1.03	0.74
DDC50 [$\times 10^{-3}$ mm^2/s]	72.7	72.7	72.7	72.7	72.7	≤1.16	0.65

DDC75 [$\times 10^{-3}$ mm^2/s]	81.8	54.6	68.2	64.3	75.0	≤1.36	0.55
DDC90 [$\times 10^{-3}$ mm^2/s]	18.2	100	59.1	100	55.0	≤2.32	0.50
DDC_{skw}	63.6	90.9	77.3	87.5	71.4	>0.48	0.81
DDC_{kur}	54.6	90.9	72.7	85.7	66.7	>1.04	0.69
α10	81.8	90.9	86.4	90.0	83.3	≤0.85	0.91
α25	90.9	72.7	81.8	76.9	88.9	≤0.89	0.87
α50	63.6	90.9	77.3	87.5	71.4	≤0.87	0.83
α75	72.7	81.8	77.3	80.0	75.0	≤0.91	0.79
α90	63.6	90.9	77.3	87.5	71.4	≤0.93	0.72
α_{skw}	63.6	72.7	68.2	70.0	66.7	≤ –0.74	0.62
α_{kur}	36.4	81.8	59.1	66.7	56.3	>1.79	0.55

α, heterogeneity index; *Acc*, accuracy; *ADC*, apparent diffusion coefficient; *AUC*, area under the curve; *D*, true-diffusion coefficient; *DDC*, distributed-diffusion coefficient; *D**, pseudo-diffusion coefficient; *f*, perfusion fraction; *kur*, kurtosis; *NPV*, negative predictive value; *PPV*, positive predictive value; *Sen*, sensitivity; *skw*, skewness; *Spe*, specificity

Online Resource 4. Comparison of parameters between high- (N = 55) and low-grade diffuse gliomas

**Parameters**	**High-grade glioma**	**Low-grade glioma**	***P*-value**
ADC10 [$\times 10^{-3}$ mm^2/s]	0.95 (089–1.01)	1.21 (1.02–1.40)	0.0047[a]
D10 [$\times 10^{-3}$ mm^2/s]	0.91 (0.85–0.97)	1.19 (0.99–1.38)	0.0035[a]
DDC10 [$\times 10^{-3}$ mm^2/s]	0.95 (0.88–1.02)	1.24 (1.02–1.45)	0.0067[a]
α10	0.82 (0.80–0.84)	0.88 (0.86–0.91)	0.0020[a]
D*10 [$\times 10^{-1}$ mm^2/s]	0.05 (0.04–0.06)	0.06 (0.04–0.08)	0.1566 [a]
f90 [%]	12.8 (11.3–14.3)	7.1 (6.0–8.3)	0<.0001[a]

Data are expressed as mean values and 95% confidence intervals.

α, heterogeneity index; ADC, apparent diffusion coefficient; D, true-diffusion coefficient; D*, pseudo-diffusion; DDC, distributed-diffusion coefficient; f, perfusion fraction

[a]Mann-Whitney U-test

Online Resource 5. Diagnostic performance of parameters in differentiating between high- (N = 55) and low-grade diffuse gliomas

**Parameters**	**Sensitivity [%]**	**Specificity [%]**	**Accuracy [%]**	**Positive Predictive Value [%]**	**Negative Predictive Value [%]**	**Cutoff value**	**AUC**
ADC10 [$\times 10^{-3}$ mm^2/s]	65.5	81.8	68.2	94.7	32.1	≤0.97	0.765
D10 [$\times 10^{-3}$ mm^2/s]	69.1	81.8	71.2	95.0	34.6	≤0.93	0.774
D*10 [$\times 10^{-1}$ mm^2/s]	30.9	100	42.4	100	22.5	>0.02	0.636
f90 [%]	87.3	90.9	87.9	98.0	58.8	>7.9	0.882
DDC10 [$\times 10^{-3}$ mm^2/s]	67.3	81.8	69.7	94.9	33.3	≤0.98	0.755
α10	65.5	90.9	69.7	97.3	34.5	≤0.85	0.788
D10 + f90	81.8	90.9	83.3	97.8	50	≤0.83, >8.0	0.878

DDC10 + α10	81.8	72.7	80.3	93.8	44.4	≤0.93, ≤0.89	0.798

α, heterogeneity index; ADC, apparent diffusion coefficient; AUC, area under the curve; D, true-diffusion coefficient; D*, pseudo-diffusion coefficient; DDC, distributed-diffusion coefficient; f, perfusion fraction
